# Differential protein folding and chemical changes in lung tissues exposed to asbestos or particulates

**DOI:** 10.1038/srep12129

**Published:** 2015-07-10

**Authors:** Lorella Pascolo, Violetta Borelli, Vincenzo Canzonieri, Alessandra Gianoncelli, Giovanni Birarda, Diana E. Bedolla, Murielle Salomé, Lisa Vaccari, Carla Calligaro, Marine Cotte, Bernhard Hesse, Fernando Luisi, Giuliano Zabucchi, Mauro Melato, Clara Rizzardi

**Affiliations:** 1 Institute for Maternal and Child Health, IRCCS Burlo Garofolo, Trieste, Italy; 2 Department of Life Science, University of Trieste, Italy; 3 Division of Pathology, CRO Centro di Riferimento Oncologico, National Cancer Institute, IRCCS, Aviano (PN) Italy; 4 Elettra - Sincrotrone Trieste, Trieste, Italy; 5 Lawrence Berkeley National Laboratory, Berkeley, CA, USA; 6 Physics Department, University of Trieste, Trieste, Italy; 7 European Synchrotron Radiation Facility, Grenoble Cedex 9, France; 8 Servizio Diagnostica Veterinaria, University of Udine, Italy; 9 Sovrintendenza Medica Regionale, Inail, Trieste, Italy; 10 Department of Medical, Surgical, and Health Sciences, University of Trieste, Italy

## Abstract

Environmental and occupational inhalants may induce a large number of pulmonary diseases, with asbestos exposure being the most risky. The mechanisms are clearly related to chemical composition and physical and surface properties of materials. A combination of X-ray fluorescence (μXRF) and Fourier Transform InfraRed (μFTIR) microscopy was used to chemically characterize and compare asbestos bodies versus environmental particulates (anthracosis) in lung tissues from asbestos exposed and control patients. μXRF analyses revealed heterogeneously aggregated particles in the anthracotic structures, containing mainly Si, K, Al and Fe. Both asbestos and particulates alter lung iron homeostasis, with a more marked effect in asbestos exposure. μFTIR analyses revealed abundant proteins on asbestos bodies but not on anthracotic particles. Most importantly, the analyses demonstrated that the asbestos coating proteins contain high levels of β-sheet structures. The occurrence of conformational changes in the proteic component of the asbestos coating provides new insights into long-term asbestos effects.

A large number of pulmonary diseases are induced by the effects of nano- and micro- particulates in the inhalants, in particular cigarette smoke, environmental pollution or as a consequence of occupational exposure. With regard to occupational exposure diseases, an improvement has been noted after the introduction of severe industrial hygiene regulations in developed countries, that led to a decline in the incidence and prevalence of pneumoconiosis. However, asbestos-related diseases, particularly mesothelioma, are still a major public health problem, due to the extremely long latency period. Pneumoconiosis is a group of occupational lung diseases caused by inhaled dust particles and fibres, which causes inflammation of the pulmonary parenchyma leading to fibrosis. The most common types of pneumoconiosis include silicosis, coal workers’ pneumoconiosis and asbestosis. Asbestosis is a chronic inflammation process caused by the inhalation of excessive amounts of asbestos fibres. Pathologically, it is characterized by both a particular pattern of interstitial pulmonary fibrosis and the presence of asbestos bodies, which are the hallmarks of asbestos exposure. The pathogenic factors involved in asbestosis and other asbestos-related diseases are extremely complex. Moreover, also in undoubted cases of occupational disease, the additional role of all the various inhaled substances that may be present in the workplace is often very difficult to determine, and even more so, the coexistence of smoking and urbanization status.

Although nonspecific, a variety of histological features other than asbestos bodies and fibres may be observed in asbestosis. Among others, a common finding in the lung parenchyma of asbestos-exposed patients is anthracosis. Anthracosis is the condition that occurs when carbon and other dust particles are found as an accumulation of black pigment in the lung tissue especially around bronchovascular bundles, in interlobular septa, and beneath the pleura and in the related lymph nodes^1^,^2^.

Carbon or coal dust pigment, as well as mineral particulates, are virtually ubiquitous air pollutants of urban life, so that such an asymptomatic accumulation can be found in varying degrees among most urban dwellers and in tobacco smokers. Anthracosis can trigger by itself an inflammatory-fibrotic process in the lung and is also considered to contribute to lung cancer^3^,^4^,^5^,^6^,^7^.

Air pollution is a complex mixture of different components and it is difficult to define a relevant exposure measure when biological mechanisms are largely unknown. It is important to note that there is a biological rationale for a carcinogenic potential of mineral and organic particulates^8^ (especially fine particles) and that with heterogeneous exposures, synergic toxicological effects must also be considered.

The assessment of toxicity and carcinogenicity of poorly soluble materials in the form of dust particles and fibres is very difficult, especially compared to drug toxicology, since their harmfulness is not defined only by the chemical composition but also by their physical nature and biopersistance. Inside the body, dust fibres and particles may undergo more complex metabolic transformations than other chemical agents. When accumulated in tissues, the surface of dust may be modified by removal or deposition of chemical elements and metals, or protein adsorption^9^.

To better address the toxicological impact arising from the progressively increasing number and doses of anthropogenic compounds and nano/ultrafine particles, it is crucial to compare the effects of materials of different nature, and relate them to the long studied mechanism driving the tissue response to asbestos exposure and correlated diseases. The aim of this work is to investigate the lung response to asbestos fibres and environmental particulates comparing the chemical features of the resulting deposits, namely asbestos bodies and black pigments, focusing on the mobilization of iron and other chemical elements, and on potential macromolecular changes in the tissues.

In previous studies, by using Synchrotron Radiation micro X-Ray Fluorescence (SR-μXRF) spectromicroscopy, we demonstrated that iron and other elements are involved in the tissue response to asbestos^10^. In particular, we revealed that the asbestos bodies are not inert structures, since they cause a continuous mobilization of iron in the surrounding tissues. Some authors demonstrated that also some other pollutants (even talc) may cause an alteration of iron homeostasis in the lung when inhaled^11^. By comparing the effects of coal on cultured cells, Zhang *et al*.^4^ found that the bioavailable iron contained in coal can induce an increment of ferritin in the cells and stimulate lipoperoxidation via free radical production and eventually induce cell death. Similar results were reported by McCunney *et al*., who suggested that the main active compound of coal inducing pneumoconiosis is iron^12^. However, as for asbestos, the iron abundance and its role in anthracosis need further clarification.

In this work, we performed SR-μXRF analyses of selected lung tissues containing both asbestos bodies and anthracotic material, as well as in lung tissues from subjects presenting only anthracosis from undefined environmental exposure. Amongst the different emerging imaging and microscopy techniques, SR-μXRF represents a very attractive approach, owing to its capability to determine and quantify the elemental composition of tissues^10^ with submicron lateral resolution^13^,^14^,^15^. Another advanced imaging technique that we applied in this work is micro Fourier Transform Infra Red (μFTIR) spectroscopy. μFTIR approaches are sensitive, label-free and non-damaging analytical tools for the characterization of biomolecules, revealing the vibrational pattern of the investigated samples. The technique is based on the absorption of IR radiation by molecules and molecular groups at specific frequency, therefore allowing the sample characterization. The most fundamental components of biological tissues are proteins, nucleic acids, carbohydrates and lipids, all of which have well-known absorption profiles in the mid IR frequency domain (4000–400 cm^−1^)^16^. Variations of shape, position and intensity of the bands characteristic of each tissue constituent in pathological samples are linked to alterations in their content and/or structure and possibly can provide important information regarding histology and pathology^17^. Specifically, two spectral bands are particularly useful for the study of proteins, the so called Amide I (∼1700–1600 cm^−1^) and Amide II (∼1580–1480 cm^−1^) bands, which arise primarily from the C = O stretching and N-H bending vibrations of the peptide backbone. The frequency of the Amide I is particularly sensitive to protein secondary structure (α-helix, β-sheet, random-coils and aggregates)^18^ and this characteristic has been exploited in numerous studies for revealing protein conformational modifications related to diseases such as Alzheimer’s^19^, Parkinson’s^20^ and Prion disorders^21^.

In the present work, we report that the correlation of XRF elemental analysis and FTIR biochemical characterization to conventional histological analyses assists in the identification of asbestos body and dust pollutants in lung tissues, namely through chemical element identification, detection of increased iron concentration and evaluation of IR absorption in the spectral regions of Amide I and II bands. In addition, we report novel information on the abundance and modification of the proteic components of the tissues exposed to pollutants, demonstrating for the first time that the asbestos body coating is composed of a high proportion of proteins with β-sheet conformation.

## Results

### Histological examinations

**Figure 1:**
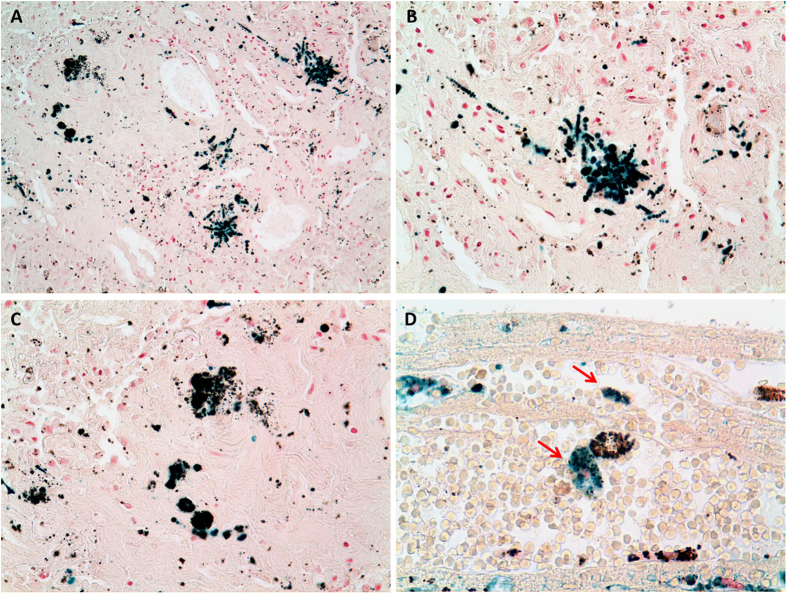
Histological images of asbestosis and anthracosis. Panel A, B, C and D are micrographs from the histological sections used in the study, coloured with Perls’ staining. Panel A (20x) shows asbestos bodies and anthracotic material embedded within asbestosis tissue. Panel B (40x), asbestos bodies strongly Perls’ positive (higher magnification of Panel A). Panel C (40x), carbonaceous particles in a patient with anthracosis (higher magnification of Panel A). Panel D, Perls’ signal in proximity of anthracosis in a patient unexposed to asbestos.

The patients with asbestosis had medium to high content of asbestos bodies in their pulmonary parenchyma, according to asbestos bodies counts performed on digested lung tissue (as reported in Table 1). We selected for the present analyses comparable lung tissue sections, having the specific histological features of asbestosis and clearly recognizable asbestos bodies. In the selected tissue slices (at least 2 per patient), the examined asbestos bodies were of various dimensions and shapes. Some of them were isolated or grouped inside dense interstitial fibrosis deposition material, others were surrounded and/or interacting with macrophages and giant cells. On many occasions, asbestos bodies were found surrounded by black pigmented material. In fact, these two types of pollutants tend to accumulate in similar regions. In the cases of only anthracosis, included in the present study, particles were concentrated around bronchovascular bundles, in interlobular septa, and beneath the pleura, being in relation to lymphatic vessel network. Dust material is dispersed, and relatively inert, causing little or no fibrosis. Only in one case is there a zonal fibrosis involving also some adjacent lymph nodes. No examples of massive fibrosis due to fibrogenic particles, which may present either as ‘coal nodules’ (of little functional significance) or as progressive massive fibrosis (which results in pulmonary function abnormalities (*coal worker’s pneumoconiosis*) were observed in our limited series. Figure 1 shows asbestos bodies compared to anthracosis after staining with the Perls’ Prussian blue method that highlights iron in the ferric state. In panel A, collections of asbestos bodies, other ferruginous bodies and deposits of carbon embedded within fibrous tissue in a case of severe pulmonary asbestosis are observed (20x). As seen in panel B at higher magnification (40x), asbestos bodies are strongly Perls positive due to the ferric iron present in their coating. Moreover, an obvious lighter blue halo surrounding the bodies is observed. In panel C, carbonaceous particles in a patient with anthracosis are shown (40x) as a collection of black granules, widely distributed in the fibrous tissue. It is generally accepted that anthracotic deposits are negative for iron stains, but in Fig. 1(D) the presence of a weakly blue stained thin outline, similar to that seen around asbestos bodies is easily observed. Panel D shows some macrophages floating in the lumen of a subpleural blood vessel of another patient with anthracosis: in the cytoplasm of one of them, indicated by arrows, black granules of coal-pigment-like material are observed along with Perls positive iron deposits (63x). Similar levels of faint positivity have been found in few macrophages and around carbon-like pigmented structures in all the tissues derived from patients presenting anthracosis.

### Elemental mapping with synchrotron XRF microscopy

**Figure 2:**
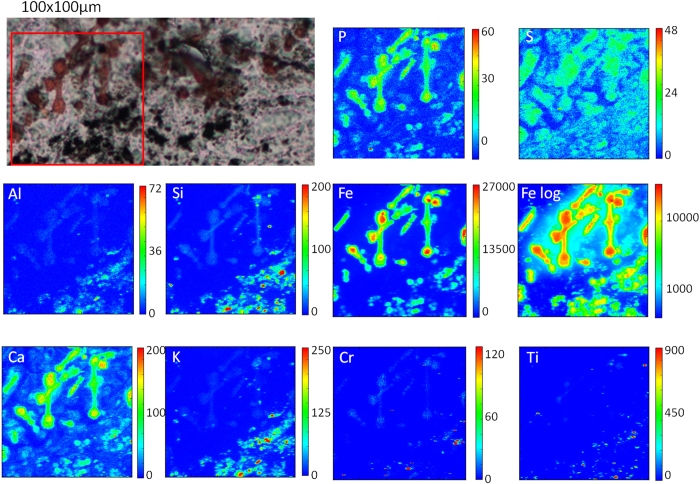
μXRF analyses of asbestos bodies and anthracosis in human lung tissue. The images show the XRF maps of P, S, Al, Si, Fe, Ca, K, Cr and Ti acquired at 7.2 keV over the area (100 μm × 100 μm) depicted in the visible light image (acquisition time 300 ms/pixel).

**Figure 3:**
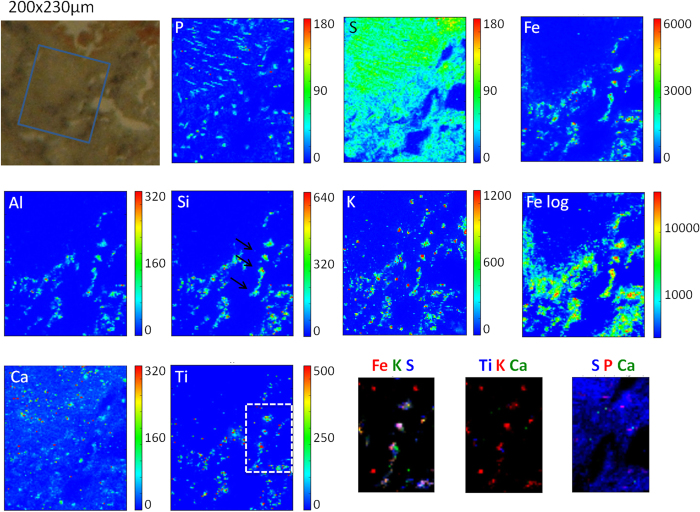
μXRF analyses of anthracosis in human lung tissue. The images show the XRF maps of P, S, Al, Si, Fe, Ca, K, Cr and Ti acquired at 7.2 keV over the area (200 μm × 300 μm) depicted in the visible light image (300 ms acquisition time/pixel). The panels in the bottom right side show the co-localisation of certain chemical elements of interest in the area indicated in the Ti map: Fe, K and S; Ti, K; and Ca; S, P and Ca, respectively.

In order to access both the lateral distribution of some light and heavy elements in the tissue samples, we performed the μXRF experiments using X-rays of 7.2 keV. The data were collected during two different beamtimes at ID21 (ESRF) from the analyses of about 20 tissue slices. The results concerning the elemental mapping for asbestos containing tissues are in agreement with our previously published results^10^,^22^. Here we report in Fig. 2 some selected results of tissue regions containing both asbestos fibres and other black anthracosis particles. The μXRF elemental maps reveal that the highest silicon (Si) concentration corresponds to the dark matter of the visible image and is thus localized inside most of it. A lower Si signal is found in association with the asbestos bodies (where Si XRF is partially masked by the high Fe levels). As revealed by the Fe maps, the maximal concentration of the element corresponds to the asbestos bodies, whilst a 5 to 10 times lower signal is revealed in the region of particulates. As highlighted by the logarithmic scale, there is a diffuse iron occurrence in the tissue structures around the asbestos bodies, as has been previously reported, suggesting a large mobilization of the element in the surrounding tissue^10^. This diffused Fe signal is also present in close proximity of the anthracotic material. Interestingly, in the anthracotic material there is an incomplete co-localization of Fe, Si and other elements, suggesting that the black discoloration is due to heterogeneous dust matter, produced by the aggregation of particles with different composition. The asbestos coating is particularly well delineated by phosphorus (P), calcium (Ca) and Fe maps^10^. The co-localization of the three elements is also present in some spots of the anthracotic material, but it is highly probable the co-localization hides the presence of unrecognizable asbestos derived structures. Sulphur (S) maps are useful to reveal morphological features of the tissue. Other chemical elements such as aluminium (Al), potassium (K), chromium (Cr) and titanium (Ti) are instead almost exclusively present in the anthracosis regions. The Al signal and very high levels of K are found to be unequivocally characteristic of these dark structures. Cr and Ti seem to belong to small particles with sizes ranging from sub-micron to tens of microns, but usually these two elements are not co-localised. Similar results to those in Fig. 2 have been obtained in different sections of lung tissue from the other patients with asbestosis (Table 1). In Fig. 3, we report a representative lung tissue section from a tissue with anthracosis only. As in the previous figure, the small black granules of anthracosis exhibit mainly Si, Fe, Al, K, Cr and Ti, in variable proportions. Still, great heterogeneity in the composition is shown by the scant co-localization of these elements. It is also interesting to note that only few black structures are identified as having significant co-localization of P and Ca, thus excluding a substantial involvement of calcification mechanisms around the anthracotic material. On the contrary, similarly to Fig. 2, the Fe map on a logarithmic scale (Fe log) confirms an increased occurrence of iron in the tissue surrounding dust material.

### μFTIR analyses on asbestos bodies and anthracosis

μFTIR analyses combined with histological observations have been performed on lung tissues deposited on different supports (see material section) and with two different set-ups, leading to comparable results.

**Figure 4:**
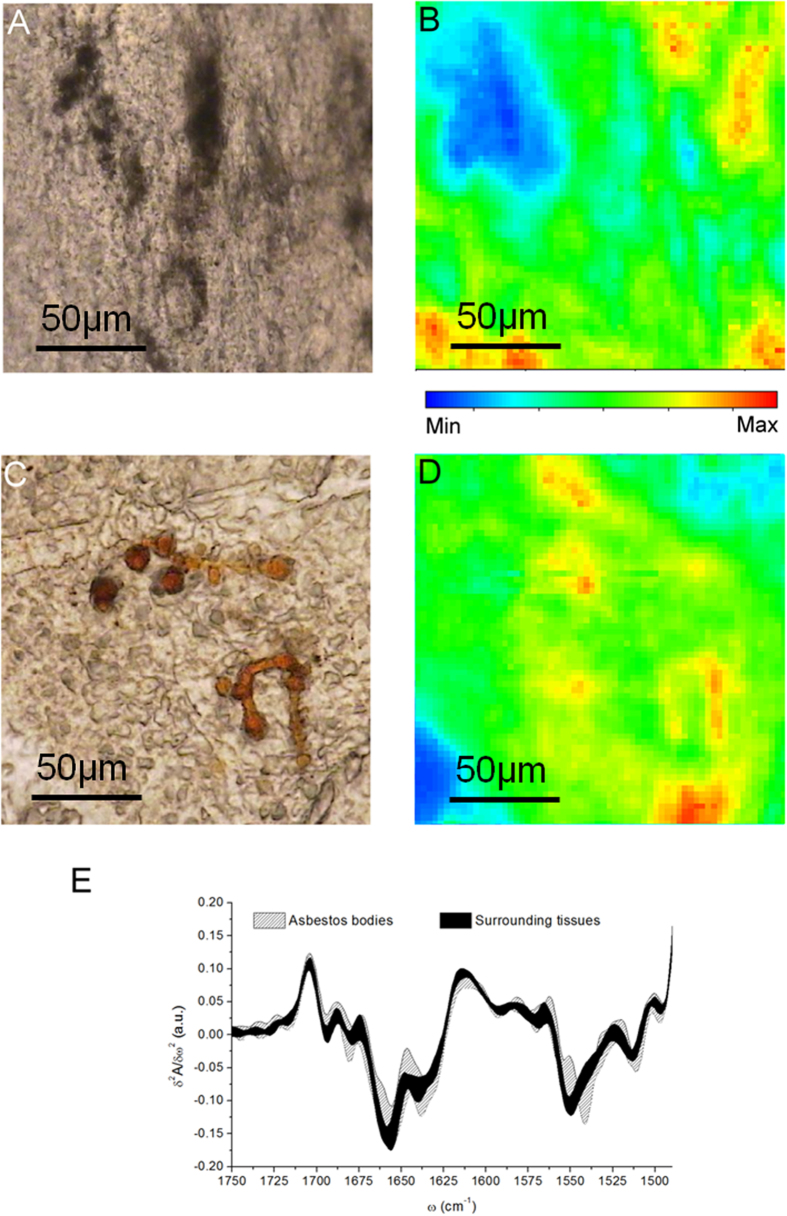
μFTIR Imaging of lung tissues with anthracosis and asbestosis. Upper row: (**A**) Optical image of the lung tissue sample deposited on ultralene film, with evidence of anthracosis; **B**) Chemical image of the 1720–1490 cm^−1^ spectral region (Amide I and II) corresponding to A. Middle row: (**C**) Optical picture of the lung tissue sample deposited on a BaF_2_ window, showing asbestos bodies; (**D**) Chemical image of the 1720–1490 cm^−1^ spectral region (Amide I and II) corresponding to C, Lower row: (**E**) Second derivative of the average vibrational spectrum of selected points on the asbestos bodies and surrounding tissues (line thickness is proportional to standard deviation).

In Fig. 4, we report an example of chemical images showing the total protein content in tissues with anthracosis (panel B) and asbestosis (panel D), generated by integrating the infrared region corresponding to protein Amide I and Amide II bands (1720–1490 cm^−1^). The protein content appears to be a significant parameter for discriminating between ferruginous bodies and carbonaceous materials, being lower and higher with respect to the surrounding tissue for anthracosis and asbestosis respectively.

However, the most intriguing result comes from the analyses of the protein’s secondary structure, which clearly reveals a local variation of the protein folding at the site of asbestos bodies. Panel E shows the average second derivative of selected spectra in the region of amide bands (line thickness is proportional to the standard deviation); the spectra were extracted from the sample regions containing asbestos bodies and from the surrounding areas. For the surrounding tissue, the Amide I band is dominated by the α-helix component centred at 1656 cm^−1^. Another component can be seen, centred at 1637 cm^−1^, possibly related to both random coiled and antiparallel β-sheet structures, as well as the 1694 cm^−1^ contribution. The standard deviation of the second derivative profile of the Amide I band at the location of ferruginous bodies is higher than that of the surrounding tissue, revealing a more heterogeneous protein pattern around the asbestos bodies. The α-helix component is still clearly visible, as well as the random coil and antiparallel β-sheet contributions; however, the strengthening of the spectral weight of the shoulders at 1629 and 1680 cm^−1^ clearly reveals the increased relative abundance of parallel β-sheet folded protein domains. The amide II profile follows the same behaviour, becoming more complex at the asbestos body sites: beyond the two components centred at 1549 and 1511 cm^−1^, a clear contribution centred at 1541 cm^−1^ is visible on the proteinaceous coating of asbestos bodies.

Overall, these results suggest that the asbestos body formation or the long time presence in lung tissue induce either the misfolding of the coating proteins or the recruitment of proteins that change conformation when interacting with the fibres.

### μFTIR analyses of extracted asbestos bodies

**Figure 5:**
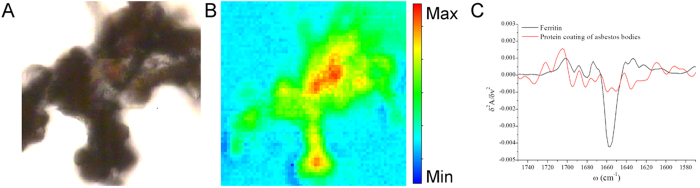
μFTIR Imaging of isolated asbestos bodies. (**A**) Optical image of extracted asbestos bodies deposited on BaF_2_ windows; (**B**) Chemical image of the 1720–1490 cm^−1^ spectral region (Amide I and II). (**C**) Second derivative of the spectrum of a selected point on the asbestos bodies (red line) and of commercial ferritin (black line).

In order to better discriminate the contribution to the protein spectral profile of the proteins firmly anchored to asbestos bodies, and to exclude the effect of fixation procedures on the evaluation of the conformational changes, we performed μFTIR analyses on asbestos bodies extracted from a frozen tissue through a mechanical procedure (Fig. 5A). The μFTIR results are exemplified in panels B and C. Panel B shows the integrated intensity of the Amide I and II bands of the freshly extracted bodies, while panel C shows the average second derivative of the spectra from the asbestos body coating compared to commercial human ferritin. The α-helix folding of ferritin is clearly deducible from the sharpness of the Amide I band centred at 1656 cm^−1^, while the β-sheet and random coiled components appears to be more abundant than that of the α–helix one for the protein coating of asbestos bodies under the experimental conditions (data not shown). Therefore, the analyses exclude the possibility that the β-sheet component could derive from specific features of ferritin, which is the major proteic component of the asbestos body coating. We could hypothesize that the ferritin molecules deposited on asbestos fibres may misfold and assume a folding pattern rich in β-sheet domains.

### Synchrotron μFTIR analyses of asbestos bodies

**Figure 6:**
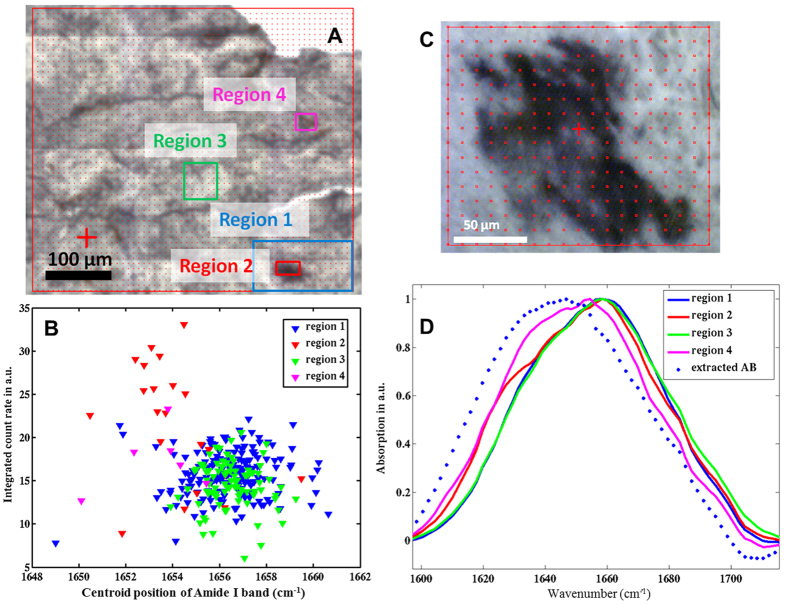
Synchrotron-FTIR map of asbestos bodies in tissue and paraffin-embedded. (**A**) Optical image of lung tissue; (**B**) Correlation plot of the integrated count rates and centroid positions of Amide I band (1720–1595 cm^−1^) of each spectrum for the four regions highlighted in Panel A. (**C**) Optical image of extracted bodies included in paraffin. (**D**) Spectral shape of Amide 1 band of the four tissue regions compared with extracted asbestos bodies (representative spectra)(Panel B).

The results of Figs 4 and 5 have been confirmed by the analyses based on measurements performed with a synchrotron source on asbestos bodies containing tissues and on samples of extracted asbestos bodies embedded in paraffin. Figure 6A shows the area which was mapped over a thin section of samples from patient A1 of Table 1. Over this map four regions were selected, for extracting spectra. The light microscope image reveals asbestos bodies in Region 2 and Region 4. Panel B in Fig. 6 shows the correlation between integrated count rate and centroid position of the Amide 1 band in the range 1720–1595 cm^−1^ from these four regions. The centroid positions of Amide 1 band of the spectra collected in regions 2 and 4 clearly tend toward lower wavenumbers, compared to regions 1 and 3 that are free from asbestos bodies. The results confirm a higher concentration of β-sheet versus α–helix on the asbestos coating. In panel D the spectral features of the Amide 1 region for the four regions of the tissue are compared with the analyses performed on extracted asbestos bodies when embedded in paraffin (panel C). As in Fig. 5, the analyses of extracted bodies reveal the highest concentration of the β-sheet component, in this case excluding the effect from inclusion in paraffin.

## Discussion

**Figure 7:**
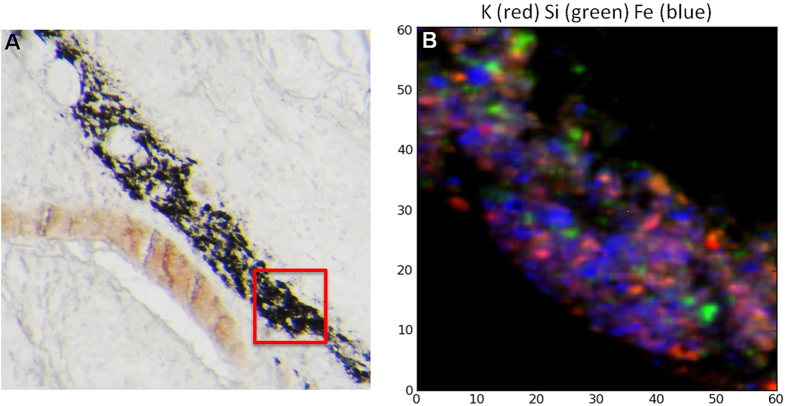
μXRF analyses of anthracosis in human lung tissue. (**A**) Optical image of an unstained lung tissue section with anthracotic material. (**B**) μXRF co-localisation of K (red), Si (green) and Fe (blue) in the red region highlighted in Panel A.

The results presented in this study aim to contribute to the understanding of lung tissue response to asbestos fibres exposure compared to environmental dust causing the presence respectively of asbestos bodies and asymptomatic anthracosis as distinctive histological features. By correlating two advanced spectroscopic techniques, namely μXRF and μFTIR, we revealed both elemental and chemical similarities and differences linked to the bio-persistence of the poorly soluble materials. Our μXRF analyses in the anthracotic structures of lung samples from patients exposed to generic urban pollution revealed high levels of Si, K and Al, together with significant Fe concentrations and traces of Cr and Ti. It is interesting that the K distribution, followed by that of Al, is the one that best matches the dark pigmentation observed in the corresponding visible light images (Figs 2 and 3). This happens both when anthracosis is found in proximity to asbestos bodies or in patients not exposed to asbestos. Potassium occurrence in anthracosis is most likely related to the presence of this element in many inhaled pollutants, although an endogenous contribution could not be excluded *a priori*. When attempting to merge the different elemental distributions, it appears that there is an incomplete or poor co-localization of the different elements (Fig. 7). This is in line with the quite heterogeneous composition of the inhaled particles that seem to aggregate in rough-edged formations of various dimensions, possibly attracted by their common hydrophobic nature. Because ultrafine particles and transition metals have been postulated to be crucial in the toxicity and potential carcinogenicity of particulate air pollution, the presence and interaction of transition metals with inhaled particles deserves particular attention. In our study, we were particularly interested in the presence and distribution of Fe in anthracosis especially compared with the mechanisms of toxicity previously considered for asbestos^10^,^11^.

Asbestos body appearance in lung tissue resulting from fibre exposure is a known and widely investigated phenomenon over that last 100 years. The formation of these structures in the lung is an intriguing phenomenon that results mainly in the deposition of endogenous iron and iron containing proteins (such as ferritin), along with some other components, on the bio-persistent fibres. The locally altered homeostasis of iron produced by the reaction to asbestos fibres and the presence of the potentially reversible iron reservoir of the iron-containing proteins have a central role in the asbestos toxicity mechanism as responsible for increased iron-mediated Reactive Oxygen Species (ROS) production. In a recent study^10^, we revealed by SR-XRF microscopy that frequently around well-formed asbestos bodies there is an increased iron occurrence that can be ascribed to two possible scenarios: i) furthering of the process that results in an increased deposition of Fe-containing moieties around the body and/or ii) release of some iron species from the body and fibre due to possible degradation processes. Those observations are further confirmed in the present study, as exemplified in Fig. 2, where at the same time we reveal that anthracosis is another important source of iron in the lung. Interestingly, conventional analyses, such as the Perls’ staining, are poorly effective in revealing the presence of iron in anthracosis, quite similar to what happens for the Fe contained in naked asbestos fibres. For this reason, we can speculate that the iron we found in the black granules of anthracosis mostly originates from inhaled environmental particles, in which its occurrence is associated with the variable content of other exogenous elements such as Si, Al and K.

Notably, K and Al are nearly absent in the asbestos bodies and these two elements, together with Si, are greatly effective to track the black formations of anthracosis. However, the high levels of Fe and its significant diffuse concentration in the tissue surrounding the pigmented bodies support the idea that particulates, in general, alter the iron homeostasis in the lung. This should have a central role in inflammatory processes and diseases correlated to pollution exposure^23^.

Recently, Andujar *et al*. investigated the chemical nature (and effects) of nanoparticles in the lung of welders comparing with control subjects exposed to generic pollutants. In agreement with our results, they observed in the anthracosis of control subjects high levels of Al and Si. They also reported some iron occurrence in pollutants in the lungs of these subjects, although negligible compared to what was found in the nanoparticles of welders’ lungs. In our analyses, Fe levels found on the anthracotic formations are from 5 to 20 times lower than that on asbestos bodies, suggesting that anthracotic material has a definitely lower tendency to attract iron. Another difference in the elements associated with asbestos and dust particles consists of the distribution of Ca and P. As previously described by us and other authors, the asbestos body formation implies calcification mechanisms along with iron aggregation, and in the asbestos body coating there are significant amounts of Ca, P and also Mg. In contrast, Ca and P are not abundant in anthracosis, thus excluding that the reaction to dust material directly implies calcification mechanisms.

The striking novelty of the present study comes from the μFTIR spectroscopic investigations. We found that the evaluation of the total protein contribution (as Amide I and Amide II) can discriminate asbestos bodies from dust particles. We found, in fact, a marginal protein content on the dark particles of anthracosis, while a quite high concentration is found to characterize asbestos bodies. In the latter case, this is in agreement with the well-known participation of ferritin in the asbestos body formation. The low protein level in anthracosis confirms a different chemical reactivity of the particulates (organic or inorganic).

Accordingly with the current hypothesis, ferritin deposition onto asbestos fibres is a consequence of free iron complexation onto the fibre surface. The iron complexed with asbestos silanol groups, in fact, attracts this iron transporting protein. The more iron is deposited onto the fibre, the more the coating process progresses^11^. It has been reported that also dust particles can be phagocyted by alveolar macrophages, similarly to the asbestos fibres, and that may generate ferruginous bodies. We did not observe ferruginous bodies in our patients unexposed to asbestos, however, we expect that nanoparticles that develop in ferruginous bodies, would have FTIR spectra more similar to asbestos bodies (namely high in protein content).

From the analyses of the protein components by μFTIR, we obtained an important result for the understanding of the tissue reaction to asbestos fibres. We demonstrated that proteins of the asbestos coating have a high percentage of β-folded protein domains. This was unexpected, since ferritin (the principal protein of the asbestos body coating^11^,^24^,^25^) is an α-helix protein (Fig. 5)^26^. We may speculate that β-structure folding is related to conformational changes occurring over time in the proteins surrounding asbestos fibres and bodies, possibly as a response to the long term presence of the asbestos bodies. Conversely, the protein transition to β-sheets could be a specific chemical mechanism of the asbestos body formation. This conformational change would allow a stronger interaction between the protein’s hydrophilic side-chains and the charged asbestos surface: this is analogous to what has been hypothesized from *in vitro* experiments to explain the protein conformation changes in the interaction among bovine serum albumin and chrysolite^27^. We also suggest that other interacting elements, such as calcium ions, may contribute to this process.

Independently of the hypothesis, our results can surely find a place into the general picture for the pathogenesis of many chronic lung disorders, which is poorly understood but has often been found to be associated with protein-misfolding events^28^,^29^. Some evidence indicates, in fact, that exposure to common lung irritants such as cigarette smoke, environmental pollutants, and infectious viral or bacterial agents can induce endoplasmic reticulum stress and protein misfolding^28^. Further *in vitro* investigations are needed to demonstrate and understand the protein conformation changes caused by asbestos and if other pollutants may do the same.

## Conclusions

In this study, thanks to the combination of two advanced spectromicroscopy techniques, we revealed unique elemental and chemical features that characterize tissue response to environmental fibre and particles. The results demonstrate that asbestos and dust material in general alter iron homeostasis in the lung, although the effect is more pronounced in the case of asbestos exposure. In addition, our findings are the first evidence for the occurrence of a conformational change in the proteic component of the asbestos coating: this evidence adds new elements for unravelling the link between human exposure to asbestos and long-term pulmonary effects.

## Methods

### Patients, histological sample preparation, asbestos body count

Human lung samples were derived from post-mortem examination of 8 patients: 4 patients exposed to asbestos were selected from the pathological files of the Unit of Pathology of the St. Polo Hospital of Monfalcone (Italy) and already analysed in a previous study^10^; 4 patients with anthracosis were selected from the archive files of the Unit of Pathology of CRO of Aviano (Italy). The demographic description of the 8 patients is reported in Table 1. Asbestos exposed patients were chosen from a cohort of 200 former shipyard workers exposed to asbestos and dead due to asbestos-related diseases. The chosen cases are considered representative of the group according to the following characteristics: work place and nature, exposure to asbestos, type of disease (asbestosis) clinically monitored and verified by autoptic examination. Patients with anthracosis were selected from autopsies of CRO Aviano including subjects with a history of malignant tumors others than lung cancer.

The study was approved by the ethics boards of the Faculty of Medicine of the University of Trieste and of the two involved hospitals, and the methods were carried out according to the approved relevant guidelines. Human samples consisted of tissues discarded after forensic autopsy, and were retrieved with the approval of the institution. Samples were anonymous at the date of the study, namely, while clinical diagnosis was recorded, it was impossible to retrieve personal information of the patients. Histological examination of the samples and histological diagnosis (Table 1), namely asbestosis or anthracosis, was carried out at the St. Polo Hospital of Monfalcone or at CRO hospital by some of the authors, who are pathologists. The identification of asbestos body or anthracosis was performed by light microscopy (Leica Microsystems, Germany) on 3 μm thick sections from paraffin-embedded samples of non-neoplastic lung tissue both unstained and stained with hematoxylin and eosin according to the standard protocol. For each patient (from n°1 to n°4 in Table 1) at least two sections positive for the presence of asbestos were considered for XRF and Perls’ analyses. For X-ray imaging and XRF analyses, 5 μm thick sections were cut from the selected tissue regions (patients from n°1 to n°8 in Table 1), mounted on ultralene foils (4 μm thick) and air-dried, as previously described. For FTIR analyses 5 μm thick sections from the same regions were mounted on ultralene foils, BaF_2_ or MirrIR supports.

The extraction for counting of the asbestos bodies was performed using a routine method as previously reported^22^,^30^. Counts are reported as number of bodies per gram of dry tissue (Table 1).

For the isolation of the asbestos bodies by a magnetic field (see below) we used a frozen tissue sample relative to the patient n° 9 in Table 1. The patient belongs to the same selected group of asbestos exposed patients mentioned before.

### Histological examination and Perls’ staining

For the histological analysis, 3–5 μm sections of the paraffin-embedded lung tissue were mounted on glass slides and air-dried. Samples were deparaffinized and then stained with hematoxylin and eosin according to standard procedures, or using Perls’ staining. This is characterized by a potassium ferrocyanide solution, followed by a counterstaining with neutral red. Finally, samples were analysed using a Leica Microscope (Leica Microsystems GmbH, Germany). Chemicals were purchased from Sigma-Aldrich.

### Isolation of Asbestos Bodies Using a Magnetic Field

For purification of ferruginous bodies (FB), a procedure previously developed in our laboratory was employed^31^, that exploits the retention of these particles in a magnetic field. For the experiments described herein, two modifications of the original method were introduced. First, a stronger magnetic field and second, N_2_ cavitation, were used after the first purification step. These two new steps greatly improved the asbestos body yield (in a preliminary experiment a 50% increase was observed) and allowed the elimination of most cellular debris that, frequently were associated with asbestos bodies, depending on the sample treated. Briefly, fragments of lung tissue (about 100 g wet weight) were processed as described elsewhere by using a more efficient cell separator: superMACS magnetic separator (Miltenyi Biotec SRL, Bologna, Italy) equipped with D columns (Miltenyi Biotec). Once the FB suspension retained in the magnetic field was obtained, the contaminating material associated with FB was almost completely eliminated in two further steps, namely, a 30-min incubation with 2 mg/ml DNAse type I at room temperature and a cavitation step (4 °C to 500 psi for 30 min in a N_2_ cavitation bomb; Parr Instrument Co., Moline, IL). Subsequently, the residual cell debris still associated with many asbestos bodies were almost completely eliminated by 3 washes in H_2_O_2_ at 150 × g for 5 min, as judged by optical microscopy on Diff-Quik-stained smears. As previously described, each pool of isolated FBs was examined with a scanning electron microscope equipped with energy-dispersive X-ray analysis (EDX). Borelli *et al*.^31^ reported the ultrastructural appearance of most FBs was that of asbestos bodies. The core analysis of asbestos bodies was possible only for the structures that presented at least one region having an almost denuded core fibre, revealing the presence of mostly amosite and crocidolite in a few cases (data not shown). Finally, FBs were counted in a Thoma counting chamber, re-suspended in H_2_O, and stored at −20 °C until use. The concentration of asbestos bodies in our samples (autopsy 60) was as follows: 110 × 10^6^/ml. We note that in the final preparations of FB, free asbestos fibres were completely absent. Comparison between the FB before (lung tissue and homogenate) and after purification by ultrastructural and optical analysis excluded that the isolation procedure led by itself to any change (apart from a low degree of fragmentation) in the morphology of asbestos bodies that might affect their reactivity.

Aliquots of extracted asbestos bodies were also embedded in paraffin, in order to mimic archival tissue sample conditions: for FTIR analyses 5 μm thick slices were obtained from these samples.

### Micro X-ray fluorescence at ID21

The XRF analyses were carried out at the ID21 beamline at the European Synchrotron Radiation Facility (ESRF, Grenoble, France), with the setup and conditions previously described^32^,^33^. The rejection of unwanted beam harmonics was ensured by a Ni coated silicon double mirror deflecting in the horizontal plane. A Si(111) fixed exit double crystal monochromator (Kohzu, Japan) was used to select and scan the beam energy. The 7.2 keV monochromatic X-ray beam was focused onto the sample using a Fresnel zone plate (Zone Plate Ltd, UK). The spot size was 0.2 μm × 1 μm with a photon flux of 3.5 × 10^9^ photon/s/Si(111) band width. The sample was mounted on a custom x-y-z stage, tilted by 30° from the optical axis, facing a fluorescence detector placed 3.5 cm from the sample. This detector was a silicon drift diode (Bruker, Germany) with 80 mm^2^ active area equipped with a low energy polymer window. The incident beam intensity was monitored upstream of the sample using a drilled photodiode collecting the fluorescence signal from a thin Ti coated Si_3_N_4_ membrane inserted in the beam path. A transmission silicon photodiode was placed downstream of the sample and allowed the collection of absorption images, whilst simultaneously collecting the XRF signal. Images were acquired by raster scanning the samples under the beam, with a step size from 0.25 to 2 μm. The acquisition time was typically 300 ms/pixel. Deconvolution of the XRF elemental maps was performed using PyMCA software^34^.

### Fourier Transform InfraRed (FTIR) spectromicroscopy

FTIR spectral images were recorded at two synchrotron facilities: SISSI beamline (Elettra Sincrotrone Trieste, Trieste, Italy) and ID21 beamline Synchrotron (European Synchrotron Radiation Facility, ESRF).

At the SISSI beamline, measurements were carried out with a conventional source using an interferometer Bruker Vertex 70 coupled with a Hyperion 3000 Vis-IR microscope equipped with a liquid nitrogen cooled bidimensional focal plane array (FPA) detector (64 × 64 pixels), using both 15xNA0.4 or 36X/NA0.5 Schwarzschild objective, and an XYZ motorized stage. Acquisitions were performed on 5 μm lung tissue slices of patients that presented anthracosis and/or asbestos bodies. Fixed and paraffin embedded tissues were measured in different configurations: deposited on ultralene or on BaF_2_ windows. Each image was acquired in transmission mode with 4096 spectra, averaging 64 scans for each detector pixel with a spectral resolution of 4 cm^−1^. During all measurements, the sample environment was purged with nitrogen. The background signal was taken in a region close to the sample deposited directly on BaF_2_ or in air for samples deposited on ultralene. Chemical images were processed using OPUS 6.5 software (Bruker Optics GmbH) and HyperSpec^35^. The second derivative of the spectra was calculated applying a Savitsky–Golay algorithm with 13 smoothing points. Total protein images were generated by integrating the spectral region 1720–1490 cm^−1^ after application of a straight baseline correction (baseline extremes: 1720 and 1490 cm^−1^). For anthracotic tissues, a slightly reduced integration interval was chosen in order to avoid possible artefacts from the spectral contributions of ultralene centred at 1166, 998 and 971 cm^−1^. For the evaluation of the alpha and beta contributions, these were estimated by peak height of the Amide I band at specific positions: 1656 cm^−1^ and 1627 cm^−1^ for alpha-helix and parallel beta sheet respectively. Neither BaF_2_ nor ultralene foils have interfering spectral features in the region of interest.

At ID21 spectra were recorded on a Nicolet Continuum XL microscope (Thermo Fisher Scientific) equipped with a 50 μm liquid nitrogen-cooled mercury cadmium telluride (MCT/A) detector, a 32X/NA0.65 Schwarzschild objective, a motorized knife-edge aperture, an XYZ motorized stage; and coupled to a Nicolet 5700 spectrometer (Thermo Fischer Scientific)^36^. The statistical plots shown in Fig. 6 were calculated using MatLab Software. Amide I areas (1595 to 1720 range), also called integrated count rates, were calculated for each spectrum after baseline correction. The mean spectral position of the spectral range (within 1595 and 1729) is denoted centroid position.

## Additional Information

**How to cite this article**: Pascolo, L. *et al*. Differential protein folding and chemical changes in lung tissues exposed to asbestos or particulates. *Sci. Rep*. **5**, 12129; doi: 10.1038/srep12129 (2015).

## Figures and Tables

**Table 1:** Demographic and clinical characteristics of the patients.

Patient	Age	Sex	Occupation history	Asbestos body count	Anthracosis	Pathological Diagnosis
A1	75	M	Naval shipyard worker (Monfalcone)	1,209,375	Yes	Asbestosis (grade >2)
A2	77	M	Naval shipyard worker (Monfalcone)	25,600	Yes	Asbestosis (grade 2)
A3	93	M	Naval shipyard worker (Monfalcone)	1,400	Yes	Asbestosis (grade 2)
A4	74	M	Naval shipyard worker (Monfalcone)	2,700	Yes	Asbestosis (grade 2)
N1	73	M	No data		Yes	Gastric cancer
N2	75	F	No data		Yes	Breast cancer
N3	77	M	No data		Yes	Colon cancer
N4	71	M	No data		Yes Lung fibrosis	Renal cancer
Autopsy n. 60	69	M	Naval shipyard worker (Monfalcone)	110,000	ND	Asbestosis (grade >2)

ND, not determined.
